# Does GPs' self-perception of their professional role correspond to their social self-image? - A qualitative study from Germany

**DOI:** 10.1186/1471-2296-11-10

**Published:** 2010-02-04

**Authors:** Iris Natanzon, Dominik Ose, Joachim Szecsenyi, Stephen Campbell, Marco Roos, Stefanie Joos

**Affiliations:** 1 Competence Centre of General Practice, Department of General Practice and Health Services Research, University Hospital of Heidelberg, Vossstrasse 2, 69115 Heidelberg, Germany; 2 National Primary Care Research and Development Centre, University of Manchester, 5th Floor, Williamson Building, Manchester M13 9PL, UK

## Abstract

**Background:**

There is a decline in the relative numbers of general practitioners in Germany. Earlier research showed that the professional relationship between general practitioners and specialists is overshadowed by conflicts which could influence medical students not to choose a career in general practice. The aim of the study is to analyse potential discrepancies between general practitioners' self-perception of their professional role and their social self-image in relation to medical specialists and to identify potential barriers that might prevent medical students from becoming a general practitioner.

**Methods:**

A qualitative study design consisting of 16 interviews with general practitioners was chosen. Data analysis was carried out using the qualitative content analysis by Philipp Mayring.

**Results:**

There is a discrepancy between general practitioners' professional self-perception and how they perceive they are viewed by specialists. General practitioners communicate a positive self-perception of their professional role. While general practitioners think that specialists in outpatient care have a positive view on general practice, it is assessed to be negative by specialists working in hospitals and as medical teachers.

**Conclusion:**

The negatively influenced social self-image may originate particularly from "badmouthing" general practitioners at universities and in hospitals. "Badmouthing" demonstrates the importance of the consideration of psychological aspects in medical teachers and hospital specialists acting as role models. Negative comments should be considered as an important factor in influencing medical students and trainees' career choices. These aspects should be more integrated in future medical education curricula.

## Background

In Germany, especially in rural areas, the recruitment of general practitioners (GPs) is a cause for concern. It is projected that the number of GPs in Germany will decline up to 2020, while demands on primary care will rise due to an aging population [1]. This trend towards a shortfall of GPs is reinforced by the fact that the number of medical students, specialising in general practice, is also declining. In accordance with other countries, the work satisfaction of German GPs is rather low, which aggravates the recruitment problem [2-6]. This trend has been observed in competition-based healthcare systems such as Germany, Austria and Switzerland rather than in state-administered systems like Great Britain, Scandinavia or the Netherlands [7].

Due to problems in recruiting medical students and trainees for general practice in various countries, several studies have already analysed the work satisfaction level of GPs [8,9] and their image [10]. However, to further understand the problems in recruiting new GPs, it would be helpful to explore perceptions of the professional role of GPs by GPs themselves and others in the medical profession. Earlier research from Australia and UK showed that the professional relationship between GPs and specialists is overshadowed by conflicts [11] which could influence medical students' or trainees' decisions to choose a career in general practice [10]. Two varieties of perception have been defined [12]: First, the self-perception which describes individual' perception of him/herself. In this context, GPs describe their own perception of their professional role. Second, the social self-image, which describes individual' assessment of how others see them. In this context, it focuses on GPs' perceptions of how they are viewed by medical specialists.

In Germany, specialists can practice in outpatient care (ambulatory care) and in hospitals (stationary care). In 2007, approximately 61000 specialists worked in outpatient care with remuneration by the statutory health insurance and about 150000 specialists worked in stationary care [13]. This paper will describe GPs' perception in relation to three target groups: specialists in outpatient care, hospital specialists and specialists working as medical teachers.

The aim of the study was to explore GPs' self-perception of their professional role and their social self-image and to identify possible barriers that might prevent medical students from becoming a general practitioner.

## Methods

### Design of the study

A qualitative study consisting of in-depth interviews was chosen to allow an intensive analysis of subjective motives, attitudes and needs. In-depth interviews are an established qualitative research method to collect information from particular groups e.g. professional target groups. An additional strength of the qualitative approach for this specific study is the chance to obtain a more realistic feel of self-perception and social self-image that cannot be experienced in the numerical data and statistical analysis used in quantitative research.

### Sample

In April 2008 letters of invitation to participate in the study were sent to a sample of 160 GPs. Addresses were randomly chosen from a symposium member database including about 800 addresses of GPs in the area of Baden-Wuerttemberg. Furthermore, information leaflets were distributed to 200 GPs taking part in a symposium in May 2008 organized by the Department of General Practice and Health Service Research at the University Hospital of Heidelberg This symposium ("Tag der Allgemeinmedizin") takes place twice a year and is an independent event, i.e. not sponsored by the pharmaceutical industry offering continuous medical education for GPs [14]. Participation in the symposium is certified by the medical chamber of Baden-Wuerttemberg.

Based on these ways of recruitment 20 GPs expressed their interest in taking part in the study. Out of those 20 GPs, a mixed sample of 16 GPs was selected in terms of gender, age, number of years in practice, urban or rural setting, solo practice or group practice. Individual appointments for the interviews were arranged. The sample of 16 interviewees is summarized in table 1:

**Table 1:** Sample of participating GPs (N = 16)

Gender	
female	n = 7

male	n = 9

**Age**	mean = 49,3 years
	min. 37 years, max. 66 years

**Number of years in practice**	8 GPs < 10 years
	8 GPs > 10 years

**Group practice**	n = 9

**Solo practice**	n = 7

**Rural area (< 5000 habitants)**	n = 4

**Medium-sized town (5000-20000 habitants)**	n = 4

**Town (> 20000 habitants)**	n = 8

### Data collection

The in-depth interviews were undertaken in the practice of the GP or in the Department of General Practice and Health Service Research, University Hospital of Heidelberg, Germany. The interviews were semi-structured and conducted by a sociologist (IN), who is experienced in conducting interviews. Each interview lasted between 45-60 minutes. All interviews were recorded digitally and transcribed verbatim.

The interviews were based on the following questions:

How do you think medical specialists perceive you as a GP? 

Do you agree with this perception of specialists? 

How would you describe yourself in your role as a GP? 

The aims of the study were explained to each interviewee. The interviewer ensured that each aspect of these questions was explained sufficiently, so that no questions or misunderstandings remained.

### Ethics approval

The ethics committee of the Heidelberg Medical School informed us that approval by an ethics committee was not necessary for a study which does not involves patient data.

### Data analysis

The interviews were carried out between May and July 2008. Analyses were carried out using the software ATLAS.ti. Key issues were identified, summarized, labelled as codes and sorted into main and sub-categories based on the qualitative content analysis by P. Mayring [15]. The aspects of interpretation and categories are developed near to the material. Using this approach, qualitative content analysis has developed procedures of inductive category development. Each category was attributed to a quotation [16]. The interviews and the analyses were conducted simultaneously, so that the researchers could control for topic saturation. Topic saturation occurred after the 13^th ^interview. I.N. and S.J. independently reviewed transcripts to confirm that the codes were comprehensive and reproducible. Disagreements during this process were discussed until a consensus was achieved. The quotations cited here were translated by IN from German into English and cross-checked by SJ.

## Results

The following main categories were identified from the transcripts: 'Impression', 'Reasons' and 'Positive self-perception'.

### Impression (GPs' social self-image)

Following sub categories were defined for the main category "Impression" (see table 2):

**Table 2:** Sub categories for the main category „Impression“

Main category	Sub category
**C1: Impression**	
	
**C1.1**: GPs think that specialists in outpatient care have a positive view on GPs	• Good cooperation with specialists in outpatient care

**C1.2**: GPs think that hospital specialists and medical teachers have a negative view on GPs	• Impression of lower respect of GPs' profession by hospital specialists
	• Badmouthing GPs by hospital specialists and medical teachers

The interviews showed a difference in GPs' views of how they are viewed by specialists who work in outpatient care, hospital specialists and medical teachers: GPs suggested that specialists who work in a hospital setting or in universities have a predominantly negative view of GPs whereas specialists who work in outpatient settings have a positive view of GPs.

#### • Good cooperation with specialists in outpatient care

Most interviewees described a feeling of a good cooperation with specialists in outpatient care.

"I'm not sure but I think that we have a good standing because we have a good relationship [with outpatient care specialists] and the cooperation is also good." (GP 3) 

#### • Impression of lower respect by hospital specialists

In contrast, the impression was negative when GPs were asked about hospital specialists. Most interviewees had the impression that there is a lack of recognition and that the professional role of GPs is not respected by hospital specialists.

"For them, general practice is something for a loser." (GP 6)

The interviewees described how hospital specialists differentiate between the level of their own work and the work of GPs, which specialists are perceived to think is less demanding.

"From their point of view, GPs can't do anything right (...) GPs are just gatekeepers. Specialists don't consider that our vocational training also needs 5 years." (GP 5) 

#### • Badmouthing GPs by hospital specialists and medical teachers

These views were generated from interviewees' experiences in hospitals during their vocational training. Especially interviewees with a shorter number of years in practice (1-5 years in practice) noted that GPs often were "badmouthed" by specialists working in hospital.

"During my clinical year I observed that specialists speak negatively about GPs' work." (GP 15) 

"When I explained during my medical training in hospital that I would like to become a GP, they laughed about my ambition."(GP 8) 

Additionally, medical teachers from other specialities than general practice at universities were perceived by interviewees to talk negatively about general practice. This was seen as having a negative influence on medical students' views regarding general practice.

"As a medical student you get the impression from the professors that GPs don't know what they are doing." (GP 11) 

"Specialists who teach at universities don't have a good opinion about GPs. This opinion could influence the medical trainees during their study." (GP 8) 

The reasons for GPs' assumption of how hospital specialists and medical teachers see them are relevant for understanding why most of the GPs describe their social self-image in relation to hospital specialists and medical teachers as predominantly negative.

### Reasons (Reason for GPs' assumption)

Following sub categories were defined for "Reasons" (see table 3):

**Table 3:** Sub category for the main category „Reasons"

Main category	Sub category
**C1: Reasons**	• Lack of specialisation
	• Lower income
	• Low quality of referrals
	• Lack of technical-oriented skills
	• Old-fashioned profession

#### • Lack of specialisation

From GPs' point of view, specialists see themselves as more qualified than GPs due to their specialisation and technical skills.

"Specialists have other skills, they take training which is more technically orientated." (GP 16) 

#### • Lower income

GPs suggested that the lower income of GPs compared to that of specialists may be seen as an indicator for a less qualified work and less prestige by many specialists.

„Maybe the income...specialists get a better income than us, this could be a quality aspect." (GP 11) 

#### • Low quality of referrals

Referrals are an important connector between specialists' and GPs' work. According to GPs' opinion, referrals are also a relevant factor in building specialists' opinions about GPs. If the quality of referrals is low, this might reinforce specialists' negative opinion of GPs' work.

„There are a few GPs, who don't perform well professionally. I observed it during my work at hospital: the referrals were disastrous." (GP 11) 

#### • Lack of technical-oriented skills

Many of the GPs stated that communication skills and problem solving skills, which are seen as crucial skills for GPs, were perceived to be of less importance for specialists who prioritised technical-oriented skills.

„Of course we are good regarding patient management, but regarding therapy and diagnosis our efforts are rather looked down on. " (GP 12) 

#### • Old-fashioned profession

In general, interviewees reported that hospital specialists see GPs like 'country doctors' having an old-fashioned way of diagnosing and treating a disease.

„I think they see us like a traditional country doctor with rather limited knowledge and seem rather ridiculous." (GP 10) 

To get an impression about GPs' self-perception the following section will describe their self-perception regarding their professional role.

### Positive self-perception (GPs' self-perception of their professional role)

Following sub categories were defined for "Positive self-perception" (see table 4):

**Table 4:** Sub category for the main category „Positive self-perception"

Main category	Sub category
**C1: Positive self-perception**	
	
C1.1: High importance of GPs' profession	• Coordinator of patient care
	• Same authority like specialists but different focus
	• Focus on psycho-social aspects
	• Wide spectrum of tasks
	• High level of responsibility

#### • Coordinator of patient care

Most of the interviewees reported a positive self-perception regarding their professional role. They were proud and self-confident due to their multi-faceted role.

In general, the interviewees felt that they play a significant role in patient care as first contact and coordinator of patient care.

„You have insights in different fields of medicine and that's the reason why I work in this field. You have a wider horizon in every way than specialists. That's why I don't agree with specialists' point of view." (GP 5) 

"I have the function of an organiser and coordinator who keeps an overview." (GP 16) 

#### • Same authority like specialists

Most interviewees explained that they have the same authority as specialists but a different focus requiring different core competencies.

#### • Focus on psycho-social aspects

GPs see themselves as doctors with a holistic perspective focusing on the psycho-social background of patients.

"I have been working as a GP for 20 years. I realise if a patient has a psychological problem or not. I mean, that's very important!" (GP 6) 

#### • Wide spectrum of tasks

#### • High level of responsibility

Interviewees explained that their work includes a wide spectrum of tasks with a high level of responsibility. In their opinion, GPs' work deserves more respect and recognition from specialists.

"It's a job which includes responsible tasks. But we don't receive any recognition from the specialists." (GP3) 

## Discussion

### Key findings

Our findings show discrepancies between GPs' self-perception of their professional role and their social self-image in relation to medical specialists. Generally, this study found that GPs have a positive self-perception regarding their professional role. They are satisfied with their multi-faceted work and its associated level of responsibility. While GPs' social self-image is perceived as being positive by specialists in outpatient care, it is negative for specialists working in hospitals and for medical teachers. The negatively social self-image originates from different factors like lower income of GPs, different skills and core competencies.

### Strengths and limitations

The study includes a mixed sample which is balanced regarding the most relevant demographic characteristics of German GPs (gender, age, solo or group practice, GPs from village, medium-sized town and town). However, the study was undertaken in only one region of Germany and the findings may not be transferable to other regions of Germany. A further strength of the study is the trustful ambiance during the conduction of the interviews with authentic reports about opinions and experiences of the interviewees. The different perspectives from varied researchers' professions (sociologist/physician) increase the intersubjective traceability.

A limitation of the study may be seen in the low number of 20 volunteers. However, out of the 20 volunteers it was possible to assemble a well-balanced sample in terms of gender, age, practice experience and practice site. Thus, compared to other interview studies and due to the fact that topic saturation occured after the 13^th ^interview, 16 interviews seem to be sufficient for an in-depth exploration of our research question.

A further limitation of the study seems to be that the interviews only present the point of view of GPs about their own profession. However, for a detailed and profound understanding it is important to analyse one perspective in a first step. For our main research question "*Does the self-perception of GPs' professional role correspondent to their social self-image *" the GPs' viewpoint is most relevant. In a next step it could be explored whether the social self-image of GPs equates to specialists perception about GPs' professional role. Therefore, specialists could be interviewed. This two-step approach is comparable to preceding qualitative studies from the Netherlands about specialists' and GPs' perceptions [17,18].

### Comparison with existing literature

We know from sociological research that it is important for everyone to create a positive self-perception and to maintain it (Theory of Appreciation) [12]. It is a pernicious experience to learn that self-perception does not correspond with the perception of others and, moreover, that the perception of others is more negative that your own perception of yourself. Beyond the need for appreciation, everyone has a need for a correspondence between self-perception and the perception of others, which has been referred to as the Theory of Consistency [12].

Previous qualitative research with hospital specialists from the Netherlands shows that specialists would be interested in developing a better collaboration with GPs on a personal level [17]. However, barriers to developing such collaborations exist because specialists believe that there is not much they can learn from GPs. With respect to professional expertise, therefore, specialists do not consider GPs as equals. Another study from the Netherlands explored experiences of GPs with hospital specialists [18]. This study showed that GPs hope to gain respect from hospital specialists and that they were also interested in developing a personal relationship and direct communication for a better collaboration.

In comparison to the Netherlands, where specialists predominantly work in hospitals, Germany has almost half of all specialists work in outpatient care.

Our results show that one has to differentiate between these types of specialists. Specialists in outpatient care usually have good working relationships with GPs - indeed, the practice would suffer if they didn't.

A research study in Australia explored the frequency of "badmouthing" by specialists as medical teachers about metropolitan and rural GPs [10]. Medical "badmouthing" has been defined as *"unwarranted, negative and denigratory comments made by doctors about other doctors in different branches of medicine" *[10]. The underlying psychological mechanism of "badmouthing" stems from a common human need for self-aggrandisement and defining of group membership by aggressively putting down people outside the "in-group" [19].

In general, specialists working in hospitals and as medical teachers have different roles and responsibilities within their occupation. Trainee doctors and medical students learn from what their teachers say and from what they do in their clinical practice, as well teachers' comments happen by the way [20]. Insufficient communication can lead to misunderstandings and create a wrong image of other professions. That's why during medical education and vocational training specialists' opinion could influence medical students' opinion.

## Conclusion

The research studies about specialists' and GPs' relationship show us that not only in Germany, but also in other countries GPs and specialists need to understand and to respect each other's role and tasks. In a Canadian study significant differences in problem-solving approaches between GPs and the more disease-focused specialists were found. Mutual respect for these fundamental differences will lead to improve the relationship between both professions and additionally health care efficiency and effectiveness [21]. However, it requires mechanisms to enable doctors that already have a heavy workload to interact regularly with each other [22].

Future research should be targeted on specialists working in hospitals, outpatient care or as medical teachers to explore their perception of GPs and to compare their views with GPs' self-perception and social self-image in relation to medical specialists. Furthermore, medical students' and vocational trainees' opinions could be explored about general practice and their experiences during medical education and vocational training.

It would be necessary to improve communication between GPs and specialists, e.g. by establishing joint continuing education courses for GPs and specialists in a regional smaller setting; for example, using a quality circle approach. Thus, it could be possible to influence the views of specialists and to achieve an atmosphere of mutual respect within and between the professional groups. Additionally a better representation of general practice in hospitals and universities could foster students' and vocational trainees' understanding about general practice. Furthermore, "badmouthing" in hospitals and universities demonstrates the importance of the consideration of psychological aspects in medical teachers and hospital specialists acting as role models. Negative comments should not be seen as a trivial offence but considered as an important factor in influencing medical students and trainees' career choices. This could be a precondition for upgrading general practice as an attractive profession for medical students during medical training thereby improving the recruitment of GPs. All these aspects should be more integrated in future medical education curricula.

## Competing interests

The authors declare that they have no competing interests.

## Authors' contributions

IN and SJ conceived the study, IN conducted and analysed the interviews. IN drafted the manuscript. SJ, DO, SC, MR and JS were involved in the study design and made contributions to the manuscript. All authors read and approved the final manuscript.

## Pre-publication history

The pre-publication history for this paper can be accessed here:

http://www.biomedcentral.com/1471-2296/11/10/prepub

## References

[B1] Die Senatorin fürArbeit, Frauen, Gesundheit, Jugend und Soziales. Deutscher Hausärzteverbund e.V. Sicherstellung der hausärztlichen Versorgung in Deutschland Primärversorgung in Deutschland im Jahr 2020 2007 9 10

[B2] Kmietowicz Z GPs shut surgeries in protest at government targets BMJ 2001 322 7294 1082 10.1136/bmj.322.7294.1082 11337429 PMC1120229

[B3] Smith R Why are doctors so unhappy? BMJ 2001 322 7294 1073 4 10.1136/bmj.322.7294.1073 11337419 PMC1120219

[B4] McGlone SJ Chenoweth IG Job demands and control as predictors of occupational satisfaction in general practice Med J Aust 2001 175 2 88 91 11556426 10.5694/j.1326-5377.2001.tb143537.x

[B5] Chew M Williams A Australian general practitioners: Desperately seeking satisfaction Med J Aust 2001 175 2 88 1 11556425 10.5694/j.1326-5377.2001.tb143536.x

[B6] Murray A Montgomery JE Chang H Rogers WH Inui T Safran FG Doctor discontent. A comparison of physician satisfaction in different delivery system settings, 1986 and 1997 J Gen Intern Med 2001 16 7 452 9 10.1046/j.1525-1497.2001.016007452.x 11520382 PMC1495236

[B7] Wendt C Gesundheitssysteme im internationalen Vergleich. Ein Überblick über den Forschungsstand Gesundheitswesen 2006 68 593 599 10.1055/s-2006-927046 17099819

[B8] Grol R Mokkinks H Smits A Work satisfaction of general practitioners and the quality of patient Care Family Practice 1985 2 128 135 10.1093/fampra/2.3.128 4043602

[B9] Appleton K House A Dowell A A survey of job satisfaction, sources of stress and psychological symptoms among general practitioners in Leeds British Journal of General Practice 1998 48 1059 63 9624747 PMC1410027

[B10] Kamien BA Bassiri M Kamien M Doctors badmouthing each other. Does it affect medical students' career choices? Aust Fam Physician 1999 28 576 79 10399390

[B11] Marshall MN Qualitative study of educational interaction between general practitioners and specialists BMJ 1998 316 442 45 9492673 10.1136/bmj.316.7129.442 PMC2665624

[B12] Stahlmann K Ehrlichkeit Selbst- und Fremdbild Huth, Dörthe: „Ehrlichkeit im Management 2003 Verlag Dr. Müller, Düsseldorf 17 29

[B13] Kassenärztliche Bundesvereinigung Grunddaten: Ärzte, Stand 2007 http://www.kbv.de/publikationen/125.html (last access: September 15^th ^2008)

[B14] Szecsenyi J Wiesemann A Stutzke O Mahler C „Tag der Allgemeinmedizin“ - ein Beitrag zur Entwicklung einer gemeinsamen regionalen Plattform zwischen Hausarztpraxen und Universitätsabteilungen Z Allg Med 2006 82 449 455 10.1055/s-2006-942191

[B15] Mayring P Qualitative Inhaltsanalyse Grundlagen und Techniken 2008 Beltz Verlag

[B16] Krippendorff K Content analysis An introduction to its methodology 1980 Beverly Hills: Sage

[B17] Berendsen A Benneker W Schuling J Rijkers-Koorn N Slaets J Meyboom-de Jong B Collaboration with general practitioners: preferences of medical specialists - a qualitative study BMC Health Services Research 2006 6 155 10.1186/1472-6963-6-155 17144921 PMC1698481

[B18] Berendsen A Benneker W Meyboom-de Jong B Klazinga N Schuling J Motives and preferences of general practitioners for new collaboration models with medical specialists: a qualitative study BMC Health Services Research 2007 7 4 10.1186/1472-6963-7-4 17207278 PMC1774564

[B19] Peach HG Badmouthing between disciplines Aus Fam Physician 1999 28 581 10399391

[B20] Harden RM Crosby JR The good teacher is more than a lecturer - twelve roles of a teacher Medical Teacher 2000 22 4

[B21] Rosser WW Approach to diagnosis by primary care clinicians and specialists: is there a difference? Journal of Family Practice 1996 42 2 139 44 8606303

[B22] Kamien M Badmouthing GPs The Medical Journal of Australia 2003 179 9 509 510 14583088 10.5694/j.1326-5377.2003.tb05660.x

